# Research on a New Model for Predicting Vault After Implantable Collamer Lens (ICL) Based on Structural Parameters of the Posterior Chamber

**DOI:** 10.1155/joph/5298174

**Published:** 2026-10-06

**Authors:** Qin Wang, Rundong Yang, Qizhi Zhou

**Affiliations:** ^1^ Department of Refractive Surgery Chongqing Aier Eye Hospital Aier Eye Hospital Group Chongqing China aierchina.com; ^2^ Department of Refractive Surgery Chongqing Eye and Vision Care Hospital Aier Eye Hospital Group Chongqing China aierchina.com; ^3^ Aier Eye Hospital Jinan University Guangzhou Guangdong China jnu.edu.cn

**Keywords:** implantable collamer lens (ICL) implantation, posterior chamber structure, trabecular-ciliary process angle (TCA), vault, vault prediction model

## Abstract

**Objective:**

To fit a new prediction model of the implantable collamer lens (ICL) vault including the parameters of the posterior chamber structure and to validate the accuracy of the new model.

**Methods:**

Seventy patients (92 eyes) undergoing ICL implantation were enrolled in the prediction model group. Spearman’s rank correlation was used for univariate analysis to assess the relationship between postoperative vault and preoperative ocular parameters. A vault prediction model was developed using multiple linear regression. An independent validation cohort comprised 140 patients (204 eyes) who received ICL implantation. All patients completed preoperative ocular examinations. Postoperative vault was measured at 1 month. We calculated the proportion of eyes with optimal vault (250–1000 μm). Agreement between predicted and actual vault was assessed using (1) intraclass correlation coefficient (ICC); (2) Bland–Altman analysis (95% limits of agreement, LoA); and (3) 95% confidence intervals for mean differences.

**Results:**

The mean age of the sample included in the prediction model was 28.18 ± 7.07 years. Vault was positively correlated with spherical equivalent (SE), white‐to‐white (WTW) horizontal corneal diameter, anterior chamber depth (ACD), anterior chamber volume (ACV), horizontal sulcus‐to‐sulcus (hSTS) diameter, pupil diameter, and maximum ciliary body thickness (CBTmax) and negatively correlated with age, intraocular pressure (IOP), crystalline lens rise (CLR), and trabecular‐ciliary process angle (TCA). The vault prediction model (adjusted *R* ^2^ = 0.54) was as follows: vault (μm) = −1711.7–166.8 × ICL size + 378.6 × WTW horizontal corneal diameter + 506.8 × ACD − 11.8 × TCA. In the validation cohort, the prediction model demonstrated clinical utility with 88.2% of outcomes within the ideal vault range (250–1000 μm) and 95.1% of errors within ±500 μm. The correlation coefficient between the predicted vault and the actual vault is 0.51 (*p* < 0.01). The bias between the predicted and actual vault is 203.20 μm, with a 95% LoA of (−175.30–581.70) μm and a standard deviation (SD) of 193.10 μm; the ICC between the predicted vault and the actual vault is 0.51 (95% CI: 0.40, 0.60).

**Conclusion:**

The vault prediction model, incorporating the posterior chamber structural parameter TCA, demonstrated favorable predictive performance in an independent validation cohort. It may serve as a useful supplementary tool for ICL size selection.

## 1. Introduction

Myopia is a leading cause of visual impairment worldwide, and its prevalence continues to rise [1, 2]. The primary surgical approaches for correcting refractive errors include corneal laser surgery and implantable collamer lens (ICL) [3–5]. For patients unsuitable for corneal laser surgery, ICL is a preferable alternative as it does not involve corneal ablation and preserves the eye’s accommodative function [6]. ICL has been demonstrated to safely and effectively correct various refractive errors [7, 8]. Postoperative complications of ICL implantation primarily include elevated intraocular pressure (IOP), anterior subcapsular cataract, pigment dispersion syndrome, and corneal endothelial decompensation [9–12]. Notably, an abnormal vault has been reported to lead to a secondary surgery rate of approximately 0.8%–3.4% [6]. Therefore, minimizing the risk of an abnormal vault is crucial for ICL surgery.

Recent studies have identified key factors affecting the ICL vault, including white‐to‐white (WTW) horizontal corneal diameter, anterior chamber depth (ACD), anterior chamber width (ACW, measured between nasal and temporal scleral spurs), anterior chamber angle (ACA), angle‐to‐angle diameter (ATA), anterior chamber volume (ACV), angle opening distance at 500 μm (AOD500), and pupil diameter [9, 13–16]. The posterior chamber structure comprises the horizontal sulcus‐to‐sulcus (hSTS) diameter, anterior lens surface vault (STSL, distance from STS plane to lens surface), lens thickness (LT), crystalline lens rise (CLR), and aqueous depth (AQD) [14, 17–21]. Since the ICL is implanted in the posterior chamber with its four haptics positioned in the ciliary sulcus, the morphology and dimensions of the surrounding sulcus structures inevitably influence haptic placement, which in turn directly affects the ICL vault [22].

Ultrasound biomicroscopy (UBM) can visualize anterior segment structures, including the morphology of the posterior chamber [23]. Recent studies have focused on posterior chamber–related factors influencing the ICL vault. Chen Q et al. [24] found that patients with anteriorly positioned ciliary bodies had a higher incidence of excessive vault after ICL implantation. Yiming et al. [25] demonstrated that eyes with low vault exhibited significantly shorter ciliary process length (CPL) and smaller maximum ciliary body thickness (CBTmax) compared to high‐vault eyes, while the trabecular‐ciliary process angle (TCA), iris–ciliary angle (ICA), and ciliary sulcus width (CSW) were significantly larger in low‐vault eyes. TCA, ICA, CPL, CBTmax, CSW, and ICL haptic position all showed significant correlations with postoperative vault.

Similarly, Tan et al. [26] confirmed that ICA, TCA, CBTmax, CPL, posterior chamber area (PCA), and CLR were all correlated with the vault. Our previous study [16] further supported these significant correlations.

To reduce the incidence of the abnormal vault, preoperative ICL size selection is critical. According to STAAR Surgical’s recommendations, the current formula for ICL sizing primarily relies on WTW horizontal corneal diameter and ACD; however, this formula was derived from European ocular anatomy and uses a very limited set of parameters. Studies report that the risk of abnormal vault using this formula is approximately 20% [11, 27], and ICL sizes selected based on this formula tend to be oversized, increasing the risk of postoperative elevated IOP [28].

Consequently, researchers have developed various ICL sizing and vault prediction models that incorporate anterior and posterior segment parameters, using artificial intelligence (AI), machine learning (ML), and traditional statistical methods. However, none has yet achieved an ideal level of prediction accuracy. Although numerous studies have confirmed the correlations between ciliary body–related parameters and vault, no existing prediction model has incorporated these critical anatomical factors.

This study aims to preoperatively assess posterior chamber anatomy using UBM‐derived parameters and to integrate these measurements with established anterior segment metrics. We will develop a novel multivariate linear regression model for predicting the ICL vault based on these posterior chamber structural parameters. The accuracy of this new model will be rigorously evaluated to validate its efficacy as an alternative ICL sizing method.

## 2. Methods

### 2.1. Development of a Novel ICL Vault Prediction Model

Patients who underwent refractive surgery at Chongqing Eye and Vision Care Hospital, Aier Eye Hospital Group, from March 2021 to December 2022, was included. Inclusion criteria were as follows: (1) age between 18 and 45 years; (2) stable refractive status for more than 2 years, with annual change not exceeding −0.50D; (3) ACD ≥ 2.8 mm; (4) corneal endothelial cell count (ECD) ≥ 2000 cells/mm^2^; and (4) IOP between 10 and 21 mmHg. Exclusion criteria were as follows: (1) long‐term use of miotic drugs or presence of morphological or structural changes in the pupil; (2) any type of glaucoma, corneal lesions, active ocular inflammation, or other ocular diseases involving the retina or choroid; (3) history of ocular iris laser treatment or other ocular surgeries; (4) patients with severe systemic diseases or psychiatric disorders (such as depression or mania), or those who are pregnant or breastfeeding. By the principles of the Declaration of Helsinki, all patients provided written informed consent. This study was approved by the Ethics Committee of Chongqing Eye and Vision Care Hospital, Aier Eye Hospital Group.

All patients underwent a comprehensive ophthalmic examination. Preoperative assessments included the following: uncorrected visual acuity (UCVA), best corrected visual acuity (BCVA), spherical and cylindrical refractive error, cylinder axis, and spherical equivalent (SE); gonioscopy to evaluate the anterior chamber angle; IOP; slit‐lamp biomicroscopy; ultra‐widefield scanning laser ophthalmoscopy (SLO; Nikon, Japan) for fundus examination; B‐scan ultrasonography (Compact Touch, Quantel Medical, France) to assess the vitreous; optical coherence tomography (OCT; Carl Zeiss, Germany) to examine the optic disc and macula; and ECD measurement.

The Pentacam (OCULUS, Germany) system was used to measure WTW horizontal corneal diameter, ACD, ACV, central corneal thickness (CCT), LT, flat keratometry (K1), steep keratometry (K2), and mean keratometry (Km). Axial length (AL) was obtained using the IOL Master 500 (Carl Zeiss, Germany).

All patients underwent UBM (Tianjin Maida Medical Technology Co., LTD, China) to evaluate posterior chamber structures. Two experienced operators (LFF and YL) performed UBM examinations, conducting transverse scans along the horizontal and vertical meridians to obtain images at the vertical (6 to 12 o’clock) and horizontal (3 to 9 o’clock) axes, as well as radial scans at the 2, 4, 8, and 10 o’clock positions at the corneal limbus. Measured parameters included the following: ACD, AOD500, hSTS diameter, vSTS diameter, CLR, and CBTmax and TCA at the 2, 4, 8, and 10 o’clock positions. All measurements were performed by another experienced physician (QW) using the UBM’s built‐in linear and angular measurement tools. Each parameter was measured three times, and the average value was recorded. For CBTmax and TCA, the mean of the four positions (2, 4, 8, and 10 o’clock) was used for statistical analysis. The definition and measurement of TCA are illustrated in Figure 1.

**FIGURE 1:**
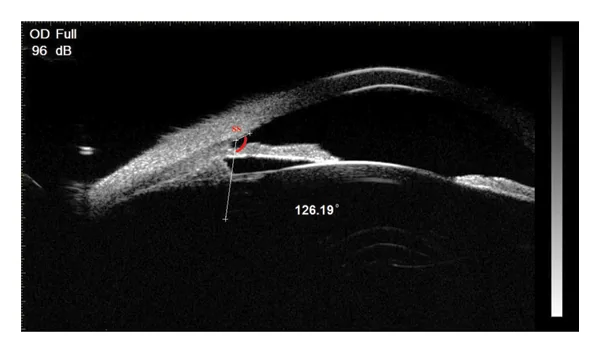
Ultrasound biomicroscopy (UBM) image showing the measurement of the trabecular‐ciliary process angle (TCA). SS, scleral spur. The red arc indicates the TCA, defined as the angle between the line connecting the scleral spur apex to the ciliary process apex and the inner wall of the sclera.

The ICL V4c size was calculated using the online formula provided by STAAR Surgical. The input parameters included WTW horizontal corneal diameter, ACD, K1, K2, and CCT. All surgical procedures were performed by the same experienced surgeon (ZQZ) following the standard surgical protocol. All implanted ICLs were positioned horizontally (3 to 9 o’clock orientation). The surgeries were successfully completed without any intraoperative complications.

Postoperative follow‐ups were conducted at 3 h, 1 day, 1 week, and 1 month to assess UCVA, BDVA, and slit‐lamp findings. Vault was additionally measured using UBM at the 1‐month visit. Subsequently, a vault prediction model was developed using stepwise multiple linear regression analysis.

### 2.2. Validation of the Novel ICL Vault Prediction Model

A total of 140 refractive error patients (204 eyes) who underwent refractive surgery at Chongqing Eye and Vision Care Hospital, Aier Eye Hospital Group, between January 2023 and October 2024, were enrolled in this study. For participant enrollment, the inclusion criteria, exclusion criteria, and preoperative examination standards and items were consistent with those previously described. By the principles of the Declaration of Helsinki, all patients provided written informed consent. All ICL surgeries were performed by the same experienced surgeon (ZQZ, as mentioned previously) following standardized protocols. All procedures were completed successfully without intraoperative complications.

All patients underwent follow‐up examinations at 3 h, 1 day, 1 week, and 1 month after ICL V4c implantation. Assessments included UCVA, BDVA, and slit‐lamp microscopy. In addition to routine examinations, UBM was performed at the 1‐month postoperative visit to measure the vault. The UBM examinations were conducted by two experienced operators (LFF and YL) using the standardized method described previously.

### 2.3. Statistical Analysis

Data analysis was conducted using SPSS Version 26.0 (IBM Corp., New York, NY, USA). Continuous variables are presented as mean ± standard deviation (mean ± SD). All continuous variables were first tested for normality using the Shapiro–Wilk test. As most variables did not follow a normal distribution, Spearman’s rank correlation coefficient was used for univariate analysis to assess the associations between preoperative parameters and postoperative vault. Variables with *p* < 0.10 in the univariate analysis were entered as candidates into a multivariate linear regression model. In addition, based on its established clinical and biomechanical importance as a fundamental determinant of postoperative vault, ICL size was forced into the model regardless of its univariate *p* value [18, 26, 29]. Multiple stepwise regression analysis was subsequently performed to generate a predictive regression equation. The agreement between the predicted and actual vault was assessed using the intraclass correlation coefficient (ICC). To evaluate the predictive accuracy of the model, the root‐mean‐square error (RMSE) and median absolute error (MedAE) were calculated. The Bland–Altman test, 95% confidence intervals (95% CI), and 95% limits of agreement (95% LoA) were used to evaluate the agreement between the predicted vault and the actual vault. *p* < 0.05 was considered statistically significant.

## 3. Results

### 3.1. ICL Vault Prediction Model

The regression model included a total of 70 patients (92 eyes) with a mean age of 28.18 ± 7.07 years. Male patients accounted for 23.91% (22/92) of the study population. Table 1 shows the baseline characteristics of the development cohort.

**TABLE 1:** Demographic and clinical characteristics of the development and validation cohorts.

Parameter	Development cohort (*n* = 92)	Validation cohort (*n* = 204)	*p* value
Age (years)	28.18 ± 7.07	28.99 ± 6.59	0.34
Sex (male, %)	23.91 (22/92)	20.10 (41/204)	0.43
SE (D)	−6.95 ± 2.37	−7.15 ± 2.53	0.04
IOP (mmHg)	15.13 ± 2.65	15.12 ± 2.54	0.42
AL (mm)	26.53 ± 1.20	26.28 ± 1.31	0.12
WTW horizontal corneal diameter (mm)	11.61 ± 0.32	11.62 ± 0.38	0.90
ACD (mm)	3.12 ± 0.25	3.15 ± 0.24	0.32
TCA (°)	121.81 ± 14.38	117.44 ± 11.06	0.01
ICL size			< 0.001
12.1 (%)	9.78	9.80	
12.6 (%)	50.00	64.22	
13.2 (%)	32.61	25.98	
13.7 (%)	7.61	1.00	

*Note:* *p* values were calculated using the independent samples *t* test for continuous variables and the chi‐square test for categorical variables. The *p* value for ICL size distribution represents the overall comparison across all four size categories.

Abbreviations: ACD, anterior chamber depth; AL, axial length; IOP, intraocular pressure; SE, spherical equivalent; TCA, trabecular‐ciliary process angle; WTW, white‐to‐white.

Table 2 shows the correlation results between parameters and vault at 1 month after ICL implantation. Parameters demonstrating significant positive correlations with vault included SE, WTW horizontal corneal diameter, ACD, ACV, hSTS diameter, pupil diameter, and CBTmax. Negative correlations were observed with age, IOP, CLR, and TCA. Based on the univariate Spearman’s correlation analysis (*p* < 0.10) and clinical considerations (ICL size was forced into the model). The multiple stepwise regression analysis results are presented in Table 3. The significant explanatory variables for vault included ICL size (*β* = −0.18, *p* = 0.05), WTW horizontal corneal diameter (*β* = 0.32, *p* = 0.005), ACD (*β* = 0.33, *p* < 0.001), and TCA (*β* = −5.33, *p* < 0.001).

**TABLE 2:** Results of correlation analysis between vault and ocular parameters at 1 month after ICL surgery.

Parameters	Spearman’s correlation	
	*R*	*p* value
Sex (male %)	24.18%	
Age (years)	−0.26	0.01
SE (D)	0.34	0.001
IOP (mmHg)	−0.23	0.03
ICL size	0.05	0.63
Km (D)	0.01	0.92
WTW horizontal corneal diameter (mm)	0.51	0.000
ACD (mm)	0.52	0.000
ACV (mm^3^)	0.29	0.04
CCT (μm)	−0.2	0.06
AL (mm)	−0.13	0.22
LT (mm)	−0.24	0.02
hSTS diameter (mm)	0.28	0.01
vSTS diameter (mm)	0.16	0.13
CLR (mm)	−0.22	0.04
Pupil diameter (mm)	0.22	0.03
CBTmax (mm)	0.36	0.000
TCA (°)	−0.56	0.000

Abbreviations: ACD, anterior chamber depth; ACV, anterior chamber volume; AL, axial length; CBTmax, maximum ciliary body thickness; CCT, central corneal thickness; CLR, crystalline lens rise; hSTS, horizontal sulcus‐to‐sulcus; IOP, intraocular pressure; LT, lens thickness; SE, spherical equivalent; TCA, trabecular‐ciliary process angle; vSTS, vertical sulcus‐to‐sulcus; WTW, white‐to‐white.

**TABLE 3:** Results of multiple stepwise regression analysis between vault and biological parameters.

Predictive parameters	Partial regression coefficient (*B*)	Standardized partial regression coefficient (*β*)	*p* value	95% CI	VIF
ICL size	−166.8	−0.18	0.05	−337.50–3.90	1.72
WTW horizontal corneal diameter (mm)	378.6	0.32	0.005	116.92–640.33	2.25
ACD (mm)	506.8	0.33	0.000	264.89–748.61	1.23
TCA (°)	−11.8	−0.43	0.000	−16.13–−7.37	1.28
Constant = −1711.7, *R* = 0.75, *R* ^2^ = 0.56, adjusted *R* ^2^ = 0.54					

Abbreviations: ACD, anterior chamber depth; TCA, trabecular‐ciliary process angle; WTW, white‐to‐white.

### 3.2. Validation Results

A total of 140 patients (204 eyes) were included in the validation group, with a mean age of 28.99 ± 6.59 years and male representation of 20.10% (41/204). The baseline characteristics of the validation cohort, along with a comparison with the development cohort, are shown in Table 1. Most baseline characteristics were comparable between the two cohorts, except for TCA (*p* = 0.01) and ICL size distribution (*p* < 0.001), which showed statistically significant differences. The actual vault measured 579.06 ± 192.38 μm, compared to the predicted vault of 767.96 ± 226.51 μm. The prediction accuracy analysis demonstrated: 68.14% (139/204) of cases had prediction errors within ±300 μm, 95.10% (194/204) of cases fell within ±500 μm of error margin, and 88.24% (180/204) of eyes achieved a clinically acceptable vault (250–1000 μm). Figure 2 illustrates the histogram of prediction errors between predicted and actual vault measurements. Figure 3 demonstrates the correlation between predicted and actual vault, revealing a statistically significant correlation coefficient of 0.51 (*p* < 0.01). Figure 4 presents the Bland–Altman analysis, which showed a mean bias (predicted–actual) of 203.20 μm with a standard deviation of 193.10 μm, and 95% LoA ranging from −175.30 to 581.70 μm. The ICC between predicted and actual vault was 0.51 (95% CI: 0.40 to 0.60). The RMSE of the model was 240.66 μm, and the MedAE was 184.06 μm.

**FIGURE 2:**
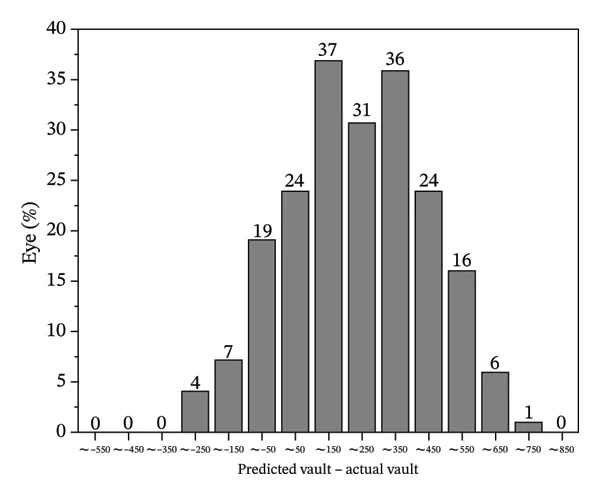
Histogram of prediction errors (predicted vault − actual vault).

**FIGURE 3:**
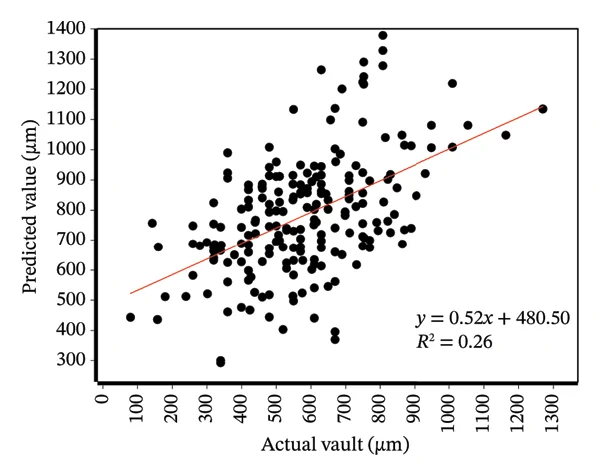
Correlation between predicted and actual vault. The solid line represents the regression line (actual vault = 480.50 + 0.52 × predicted vault, *R* ^2^ = 0.26).

**FIGURE 4:**
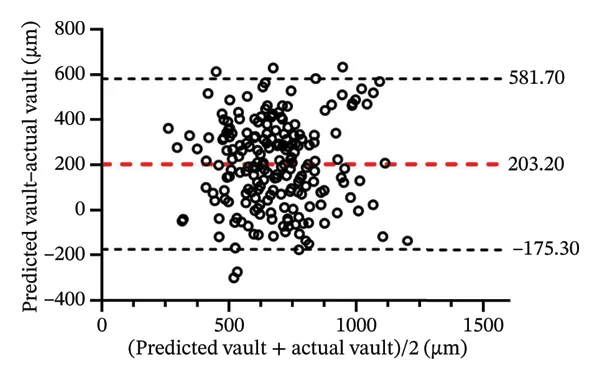
Bland–Altman plots showing the difference between the actual and predicted ICL vault. The red dashed line in the middle represents the mean differences between the actual and predicted ICL implantation vault; black dashed lines are the upper and lower borders of the 95% limits of agreement (mean difference ± 1.96 multiplied by the standard deviation of the mean difference).

## 4. Discussion

With the increasing prevalence of myopia, the number of ICL implantation procedures has been growing significantly. Vault‐related safety remains both a clinical challenge for ophthalmologists and a key research focus in the field of ophthalmology. The ultimate goal is to establish optimal ICL sizing criteria that yield an appropriate vault, thereby minimizing the need for secondary surgeries and improving clinical outcomes. The postoperative vault of ICL is affected by the anatomical characteristics of the posterior chamber. Our preliminary data demonstrate significant associations between the posterior chamber structure and vault [16]. In this study, we developed and validated a vault prediction regression model incorporating posterior chamber structural parameters. To our knowledge, this represents the first study to establish and validate an ICL vault prediction model that systematically accounts for the posterior chamber structure. The development and validation cohorts were largely comparable in baseline characteristics, supporting the generalizability of our prediction model.

Recent studies have placed increasing emphasis on the relationship between posterior chamber structures (particularly the ciliary body) and the vault. In a novel vault prediction model proposed by Reinstein et al. [30], the ciliary body inner diameter (CBID) has been demonstrated to be more highly correlated with postoperative vault than STS. Subsequently, the relationship between other posterior chamber structures and the vault has attracted increasing research attention [22]. Chen et al. [24] demonstrated that an anteriorly positioned ciliary body was associated with an increased risk of excessive vault after ICL implantation. Similarly, Yiming et al. [25] found that eyes with insufficient vault had significantly smaller CBTmax and larger TCA than those with ideal vault. These findings were corroborated by Tan et al. [26] through the linear regression analysis of ocular parameters. Our preliminary studies further revealed that vault progressively decreases as CBTmax decreases and TCA increases. In this study, we further investigated the correlations between ocular structural parameters and vault. Our results demonstrated a positive correlation between CBTmax and vault, while TCA showed a negative correlation—findings consistent with previous studies. CBTmax represents a parameter that reflects both the height and size of the ciliary body. A thicker ciliary body provides better support to the ICL haptics, ensuring their stable positioning. This anatomical support prevents the ICL from sliding toward the crystalline lens or dislocating from the ciliary sulcus, thereby minimizing the occurrence of low vault. TCA represents the positional variation in the ciliary process apex relative to the scleral spur apex. When TCA is larger, the ciliary body is positioned more anteriorly with a shallower ciliary sulcus and shorter ciliary processes. During surgery, after the ICL is placed in the posterior chamber with its haptics positioned in the ciliary sulcus, the initially unstable haptics may be shifted toward the ciliary body by continuous pupillary movement, potentially resulting in low vault [4, 31, 32]. This mechanism may explain why the post‐ICL vault tends to gradually decrease and typically stabilizes after approximately 1 year. However, further research is needed to investigate the temporal changes in ICL haptic position over time.

Kojima et al. [18] utilized UBM to measure anterior segment parameters and developed a regression model incorporating ACD, hSTS diameter, and STSL. However, the regression equation was derived from a relatively small sample size. Despite demonstrating a high *R* ^2^ value, this equation requires further validation with larger studies or expanded sample sizes for optimization. The NK formula proposed by Nakamura et al. [29] incorporated ACW and CLR as parameters (*R* ^2^ = 0.61). However, their multivariate regression analysis failed to consider other ocular parameters such as pupil size and AL. Kim et al. [21] developed a vault prediction model incorporating AQD, ACA, and LT, which demonstrated optimal Bayesian information criterion (BIC) results for postoperative ICL vault prediction. However, this regression formula did not account for other ocular parameters such as AL or corneal characteristics, nor was it externally validated. The regression model developed by Zhu et al. [14, 33] included ICL size, hSTS diameter, LT, and vSTS diameter as predictors, with an adjusted *R* ^2^ of 0.643. Although this model demonstrated robust predictive accuracy in validation studies, it did not incorporate other potentially relevant posterior chamber anatomical parameters. Tan et al. [26] established a TCA‐containing regression model (vault = 679.42 − 7.26 × TCA + 192.30 × ACD − 196.37 × CLR + 73.21 × hSTS diameter; adjusted *R* ^2^ = 0.32) without validation. In this study, we included TCA, ICL size, WTW horizontal corneal diameter, and ACD as predictors of vault, and the model achieved an adjusted *R* ^2^ of 0.54. Notably, WTW horizontal corneal diameter and ACD are also components of the STARR formula.

A point worth clarifying is the inclusion of ICL size in the final model. Although its univariate correlation with vault did not reach statistical significance (*p* = 0.63), we considered it a clinically indispensable covariate, as the physical dimension of the implanted lens is a fundamental structural determinant of the postoperative vault. This decision is consistent with previous prediction models, which also included ICL size as a core variable regardless of its univariate performance [18, 25, 29]. Notably, TCA’s independent contribution became statistically significant only after adjustment for WTW horizontal corneal diameter and ACD (*β* = −0.18, *p* = 0.05), further supporting the presence of confounding effects that were masked in the univariate analysis. This underscores the importance of incorporating clinically relevant variables based on biological plausibility rather than statistical selection alone.

In the meta‐analysis by Packer [6], selecting ICL size based on WTW horizontal corneal diameter and ACD achieved approximately 83.6% optimal vault. A study by Trancón et al. [14] demonstrated that using conventional ICL sizing methods resulted in optimal vault in 77.23% of cases. The NK1 formula, proposed by Nakamura et al. [34] based on ACW and CLR, yielded 71% normal‐vault and 23% high‐vault cases. By comparison, our model demonstrated that 88.24% (180/204) of eyes in the validation cohort achieved an optimal vault, suggesting that the inclusion of posterior chamber parameters may offer a clinically useful alternative for ICL sizing. However, it should be noted that these comparisons are derived from different study populations and should be interpreted with caution. Although slightly inferior to Zhu et al.’s [33] findings—potentially due to our smaller validation cohort—our model demonstrates strong predictive value. The predicted vault derived in this study showed a significant correlation with the actual vault (*r* = 0.51, *p* < 0.001), a result comparable to that of Kojima et al.’s [18] LASSO regression model (*r* = 0.582, *p* < 0.001), which incorporated ICL size, ACD, ACA, and LT as parameters.

Our Bland–Altman analysis revealed a systematic bias of 203.20 μm, indicating that the model tended to overestimate the postoperative vault. Several factors may contribute to this overestimation. First, TCA measurements using UBM are inherently subject to interobserver and intraobserver variability, as the angle landmarks can be subtle to identify on UBM images. Second, our model did not include other potentially important posterior chamber parameters, such as STSL (sulcus‐to‐lens distance) or CPL, which may have contributed to the prediction error. Third, the relatively small sample size in the development cohort (92 eyes) may have led to overfitting, which could reduce the model’s generalizability. Future studies with larger sample sizes and incorporation of additional anatomical parameters are warranted to further refine the model and reduce this bias.

This study has several limitations. First, while our vault prediction success rate exceeds that of the STAAR company formula, its accuracy remains relatively lower compared to other previously reported formulas, necessitating further optimization. Second, all participants in this study were of Chinese ethnicity, and anatomical variations in ciliary body characteristics among different ethnic groups may affect the generalizability of our regression equation. Finally, due to limited cases with 12.1 mm and 13.7 mm ICL sizes in our cohort, we cannot confirm the formula’s applicability for selecting these extreme ICL sizes [34].

## 5. Conclusions

In summary, this study developed and validated a novel ICL vault prediction model incorporating ICL size, WTW horizontal corneal diameter, ACD, and the posterior chamber parameter TCA. The model demonstrated favorable predictive performance in an independent validation cohort, with 88.24% of eyes achieving an optimal vault. These findings suggest that incorporating posterior chamber structural parameters may provide a useful reference for optimizing ICL size selection.

## Author Contributions

Qin Wang performed the statistical analysis and wrote the original draft, Qizhi Zhou conceptualized the study and modified the manuscript, and Rundong Yang collected data and information.

## Funding

This study was funded by the Clinic Research Foundation of Aier Eye Hospital Group (Grant No. AR2009D4).

## Disclosure

All authors read, reviewed, and approved the final manuscript.

## Ethics Statement

This prospective study was approved by the Ethical Review Committee of Chongqing Eye and Vision Care Hospital, Aier Eye Hospital Group, and it adhered to the principles of the Declaration of Helsinki.

## Consent

The authors have nothing to report.

## Conflicts of Interest

The authors declare no conflicts of interest.

## Data Availability

The data used in this study can be requested by contacting the corresponding author.

## References

[1] Holden B. A. , Fricke T. R. , Wilson D. A. et al., Global Prevalence of Myopia and High Myopia and Temporal Trends From 2000 Through 2050, Ophthalmology. (2016) 123, no. 5, 1036–1042, 10.1016/j.ophtha.2016.01.006. 26875007

[2] Zhang S. , Zhong H. , Zeng D. et al., Research Trends and Hotspots in the Relationship Between Outdoor Activities and Myopia: A Bibliometric Analysis Based on the Web of Science Database From 2006 to 2021, Frontiers in Public Health. (2020) 26, no. 10, 10.3389/fpubh.2022.1047116. PMC9644123 36388306

[3] Tanzer D. J. , Brunstetter T. , Zeber R. et al., Laser in Situ Keratomileusis in United States Naval Aviators, Journal of Cataract & Refractive Surgery. (2013) 39, no. 7, 1047–1058, 10.1016/j.jcrs.2013.01.046. 23664357

[4] Randleman J. B. , Russell B. , Ward M. A. , Thompson K. P. , and Stulting R. , Risk Factors Gnosis for Corneal Ectasia After LASIK, Ophthalmology. (2003) 110, no. 2, 267–275, 10.1016/j.ophtha.2006.03.045. 12578766

[5] Binder P. S. , Lindstrom R. L. , Stulting R. D. et al., Keratoconus and Corneal Ectasia After LASIK, Journal of Refractive Surgery. (2005) 21, no. 6, 749–752, 10.3928/1081-597X-20051101-15. 16329368

[6] Packer M. , Meta-Analysis and Review: Effectiveness, Safety, and Central Port Design of the Intraocular Collamer Lens, Clinical Ophthalmology. (2016) 1059, 10.2147/OPTH.S111620. PMC4907705 27354760

[7] Sanders D. R. , Doney K. , and Poco M. , United States Food and Drug Administration Clinical Trial of the Implantable Collamer Lens (ICL) for Moderate to High Myopia, Ophthalmology. (2004) 111, no. 9, 1683–169. 10.1016/j.ophtha.2004.03.026 15350323

[8] Wang Q. , Fan L. , and Zhou Q. , The Best Choice for Low and Moderate Myopia Patients Incapable for Corneal Refractive Surgery: Implantation of a Posterior Chamber Phakic Intraocular Lens, International Ophthalmology. (2022) 43, no. 2, 575–581, 10.1007/s10792-022-02459-3. PMC9971141 35984557

[9] Wang X. Q. , Chen X. , and Wang X. Y. , Postoperative Vault of Implantable Intraocular Lenses and Related Factors, Journal of Otolaryngology, Head and Neck Surgery of Shandong University. (2020) 34, no. 2, 72–77.

[10] Sheng X. L. , Rong W. N. , Jia Q. et al., Outcomes and Possible Risk Factors Associated with Axis Alignment and Rotational Stability After Implantation of the Toric Implantable Collamer Lens for High Myopic Astigmatism, International Journal of Ophthalmology. (2012) 5, no. 4, 459–465, 10.3980/j.issn.2222-3959.2012.04.10. PMC3428541 22937505

[11] Fernandes P. , González-Méijome J. M. , Madrid-Costa D. , Ferrer-Blasco T. , Jorge J. , and Montés-Micó R. , Implantable Collamer Posterior Chamber Intraocular Lenses: A Review of Potential Complications, Journal of Refractive Surgery. (2011) 27, no. 10, 765–776, 10.3928/1081597X-20110617-01. 21710954

[12] Kamiya K. , Shimizu K. , Kobashi H. , Igarashi A. , and Komatsu M. , Three-Year Follow-Up of Posterior Chamber Toric Phakic Intraocular Lens Implantation for Moderate to High Myopic Astigmatism, PLoS One. (2013) 8, no. 2, 10.1371/journal.pone.0056453. PMC3568037 23409187

[13] Lee H. , Kang S. Y. , Seo K. Y. et al., Dynamic Vaulting Changes in V4c Versus V4 Posterior Chamber Phakic Lenses Under Differing Lighting Conditions, American Journal of Ophthalmology. (2014) 158, no. 6, 1199–1204.e1, 10.1016/j.ajo.2014.08.020. 25149911

[14] Trancón A. S. , Manito S. C. , Sierra O. T. , Baptista A. M. , and Serra P. M. , Determining Vault Size in Implantable Collamer Lenses: Preoperative Anatomy and Lens Parameters, Journal of Cataract & Refractive Surgery. (2020) 46, no. 5, 728–736, 10.1097/j.jcrs.0000000000000146. 32358268

[15] Xiong Y. , Mao Y. Y. , and Zhang Q. , Influencing Factors of Vault After Central Canal Endoscopic Implantation of the Eye, Ophthalmology. (2020) 29, no. 6, 448–452.

[16] Wang Q. , Chen X. , Li L. et al., Effect of Anterior Segment Structure on Vault and Position After Implantable Collamer Lens Implantation, Indian Journal of Ophthalmology. (2024) 72, no. 9, 1298–1303, 10.4103/IJO.IJO_2663_23. PMC11552803 38767559

[17] Cerpa Manito S. , Sánchez Trancón A. , Torrado Sierra O. , Baptista A. M. , and Serra P. M. , Biometric and ICL-Related Risk Factors Associated to Sub-Optimal Vaults in Eyes Implanted With Implantable Collamer Lenses, Eye and Vision. (2021) 8, no. 1, 10.1186/s40662-021-00250-6. PMC8256545 34225809

[18] Kojima T. , Yokoyama S. , Ito M. et al., Optimization of an Implantable Collamer Lens Sizing Method Using High-Frequency Ultrasound Biomicroscopy, American Journal of Ophthalmology. (2012) 153, no. 4, 632–637.e1, 10.1016/j.ajo.2011.06.031. 22245462

[19] Zhang X. , Li H. Y. , and Ma B. , Analysis of the Influencing Factors of the Postoperative Vault of Posterior Chamber Intraocular Lenses in Patients With High Myopia, Medical clinical research. (2021) 38, no. 10, 1540–1543.

[20] Zhang P. , Guo C. , Wang S. , Jiang W. , Wang D. , and Yan H. , Influencing Factors Comparing Different Vault Groups After Phakic Implantable Collamer Lens Implantation: Review and Meta-Analysis, BMC Ophthalmology. (2024) 24, no. 1, 10.1186/s12886-024-03325-9. PMC10870571 38360631

[21] Kim T. , Kim S. J. , Lee B. Y. et al., Development of an Implantable Collamer Lens Sizing Model: A Retrospective Study Using ANTERION Swept-Source Optical Coherence Tomography and a Literature Review, BMC Ophthalmology. (2023) 23, no. 1, 10.1186/s12886-023-02814-7. PMC9921691 36765328

[22] Tan W. , Chen Q. , Yang R. et al., Characteristics and Factors Associated with the Position of the Haptic After ICL V4C Implantation, Journal of Cataract & Refractive Surgery. (2023) 49, no. 4, 416–422, 10.1097/j.jcrs.0000000000001134. 36700941

[23] Pavlin C. J. , Harasiewicz K. , and Foster F. S. , Ultrasound Biomicroscopy of Anterior Segment Structures in Normal and Glaucomatous Eyes, American Journal of Ophthalmology. (1992) 113, no. 4, 381–389, 10.1016/S0002-9394(14)76159-8. 1558111

[24] Chen Q. , Tan W. , Lei X. et al., Clinical Prediction of Excessive Vault After Implantable Collamer Lens Implantation Using Ciliary Body Morphology, Journal of Refractive Surgery. (2020) 36, no. 6, 380–387, 10.3928/1081597X-20200513-02. 32521025

[25] Yiming Y. , Xi C. , Huan Y. et al., Evaluation of Ciliary Body Morphology and Position of the Implantable Collamer Lens in Low-Vault Eyes Using Ultrasound Biomicroscopy, Journal of Cataract & Refractive Surgery. (2023) 49, no. 11, 1133–1139, 10.1097/j.jcrs.0000000000001285. 37586102

[26] Tan W. , Wang Z. , Zeng Q. et al., The Influence of Iris-Ciliary Angle (ICA) on the Vault After Implantation of V4c Implantable Collamer Lens: A Chain Mediation Model of ICL Haptic Related Factors, BMC Ophthalmology. (2023) 23, no. 1, 10.1186/s12886-023-03122-w. PMC10559582 37803302

[27] Lee D. H. , Choi S. H. , Chung E. S. , and Chung T. Y. , Correlation Between Preoperative Biometry and Posterior Chamber Phakic Visian Implantable Collamer Lens Vaulting, Ophthalmology. (2012) 119, no. 2, 272–277, 10.1016/j.ophtha.2011.07.047. 22054995

[28] Moshirfar M. , Placide J. , Neves da Silva H. V. et al., Assessing the Efficacy of Four Diagnostic Devices and Four Nomograms in Posterior Chamber Phakic Intraocular Lens Size Selection, Journal of Refractive Surgery. (2022) 38, no. 2, 106–111, 10.3928/1081597X-20211109-01. 35156462

[29] Nakamura T. , Isogai N. , Kojima T. , Yoshida Y. , and Sugiyama Y. , Optimization of Implantable Collamer Lens Sizing Based on Swept-Source Anterior Segment Optical Coherence Tomography, Journal of Cataract & Refractive Surgery. (2020) 46, no. 5, 742–748, 10.1097/j.jcrs.0000000000000134. 32358270

[30] Reinstein D. Z. , Archer T. J. , Vida R. S. , Piparia V. , and Potter J. G. , New Sizing Parameters and Model for Predicting Postoperative Vault for the Implantable Collamer Lens Posterior Chamber Phakic Intraocular Lens, Journal of Refractive Surgery. (2022) 38, no. 5, 272–279, 10.3928/1081597X-20220302-01. 35536711

[31] Kato S. , Shimizu K. , and Igarashi A. , Vault Changes Caused by Light-Induced Pupil Constriction and Accommodation in Eyes With an Implantable Collamer Lens, Cornea. (2019) 38, no. 2, 217–220, 10.1097/ICO.0000000000001785. PMC6344073 30371566

[32] Chen X. , Miao H. , Naidu R. K. , Wang X. , and Zhou X. , Comparison of Early Changes in and Factors Affecting Vault Following Posterior Chamber Phakic Implantable Collamer Lens Implantation Without and With a Central Hole (ICL V4 and ICL V4c), BMC Ophthalmology. (2016) 16, no. 1, 10.1186/s12886-016-0336-8. PMC5015244 27604229

[33] Zhu Q. J. , Chen W. J. , Zhu W. J. et al., Short‐Term Changes in and Preoperative Factors Affecting Vaulting After Posterior Chamber Phakic Implantable Collamer Lens Implantation, BMC Ophthalmology. (2021) 1, no. 1, 10.1186/s12886-021-01963-x. PMC8101126 33957891

[34] Nakamura T. , Isogai N. , Kojima T. , Yoshida Y. , and Sugiyama Y. , Implantable Collamer Lens-Sizing Method Based on Swept Source Anterior Segment Optical Coherence Tomography, American Journal of Ophthalmology. (2018) 187, 99–107, 10.1016/j.ajo.2017.12.015. 29294311

